# Determination of production losses related to lumpy skin disease among cattle in Turkey and analysis using SEIR epidemic model

**DOI:** 10.1186/s12917-021-02983-x

**Published:** 2021-09-07

**Authors:** Burak Mat, Mehmet Saltuk Arikan, Ahmet Cumhur Akin, Mustafa Bahadır Çevrimli, Harun Yonar, Mustafa Agah Tekindal

**Affiliations:** 1 Department of Animal Health Economics and Management, Faculty of Veterinary Medicine, Selçuk University, Konya, Turkey; 2 Department of Animal Health Economics and Management, Faculty of Veterinary Medicine, Fırat University, Elazıg, Turkey; 3 Department of Animal Health Economics and Management, Faculty of Veterinary Medicine, Mehmet Akif Ersoy University, Burdur, Turkey; 4 Department of Biostatistics, Faculty of Veterinary Medicine, Selçuk University, Konya, Turkey; 5 Department of Biostatistics, Faculty of Medicine, İzmir Katip Çelebi University, İzmir, Turkey

**Keywords:** Epidemiology, SEIR Model, Lumpy Skin Disease, production losses, Turkey

## Abstract

**Background:**

Lumpy Skin Disease (LSD) is an infectious disease induced by the Capripoxvirus, causing epidemics in Turkey and several countries worldwide and inducing significant economic losses. Although this disease occurs in Turkish cattle every year, it is a notifiable disease. In this study, LSD in Turkey was modelled using the Susceptible, Exposed, Infectious, and Recovered (SEIR) epidemiological model, and production losses were estimated with predictions of the course of the disease. The animal population was categorized into four groups: Susceptible, Exposed, Infectious, and Recovered, and model parameters were obtained. The SEIR model was formulated with an outbreak calculator simulator applied for demonstration purposes.

**Results:**

Production losses caused by the LSD epidemic and the SEIR model’s predictions on the disease’s course were evaluated. Although 1282 cases were identified in Turkey during the study period, the prevalence of LSD was calculated as 4.51%, and the mortality rate was 1.09%. The relationship between the disease duration and incubation period was emphasized in the simulated SEIR model to understand the dynamics of LSD. Early detection of the disease during the incubation period significantly affected the peak time of the disease. According to the model, if the disease was detected during the incubation period, the sick animal's time could transmit the disease (Tinf) was calculated as 2.66 days. Production loss from LSD infection was estimated at US $ 886.34 for dairy cattle and the US $ 1,066.61 for beef cattle per animal.

**Conclusion:**

Detection of LSD infection during the incubation period changes the course of the disease and may reduce the resulting economic loss.

## Background

Lumpy Skin Disease is a capripoxvirus in the family poxviridae transmitted by vectors among domestic cattle. It is a viral disease characterized by nodules, weakening of the skin, enlargement of lymph nodes, edema in the skin, and sometimes death [13, 44]. Although the disease is usually recorded in endemic areas at regular intervals, it can rapidly spread to cause epidemics in a region or a country [9].

Large-scale animal movements from East Africa to Palestine, Jordan, and Saudi Arabia occurred in 2008-2013. These animal movements have spread towards Syria, Iran, and Iraq, which share land borders with Turkey. It has been reported that there is a high level of animal activity just before the Eid Qurbani and that the risk of transmission between countries increases the risk of infectious diseases [13]. The fact that the Turkish provinces where the disease was detected in 2013 are geographically close to these countries raises the possibility of LSD coming from these countries [7].

Loss of body weight [14], spontaneous abortion (Vorster and Mapham, 2008), mastitis, decreased milk yield [42], damaged skin [20], and damaged carcasses [27] in cattle affected by the LSD cause severe production-related economic losses. Moreover, vaccination, treatment costs [42], restriction of animal movements, and eradication practices also cause significant financial losses [45].

Modelling animal epidemics' identification and dynamics contributes significantly to their control, treatment, and eradication by explaining the disease's transmission behaviour. The data provided by modelling methods during the formative phase of animal epidemics form the basis of defining useful scenarios for combating the disease. Early detection of infectious diseases and some intervention measures, combined with the temporary restriction on animals' movement, can significantly reduce the epidemic's infectivity and the adverse effects of the disease [26]. The SEIR model is widely used in modelling animal epidemics. This model has previously been used to successfully model animal epidemics such as bovine brucella [38], contagious bovine pleuropneumonia [5, 6], rabies [39], swine influenza [29], avian influenza [35] and equine influenza [33].

In this study, LSD in Turkish cattle was modelled using the SEIR epidemiological model. Production losses in dairy cattle and beef cattle were calculated together with predictions about the course of the disease.

## Results

The epidemiological findings of LSD infection in Turkey in 2019 are presented in Table 1.

**Table 1:** Number of outbreaks, cases, deceased, and destroyed animals by city

Province	Number of Outbreaks	Suspicious	Case	Dying	Destruction/Culling	Fatality Rate (%)	Prevalence (%)	Mortality (%)
Adıyaman	2	38	7	4	3	57.14	18.42	10.53
Agri	8	2266	40	14	26	35.00	1.77	0.62
Artvin	3	206	15	3	12	20.00	7.28	1.46
Diyarbakir	11	361	36	9	27	25.00	9.97	2.49
Elazig	8	422	51	7	44	13.73	12.09	1.66
Erzincan	2	175	14	1	13	7.14	8.00	0.57
Erzurum	15	7856	142	36	106	25.35	1.81	0.46
Eskisehir	1	315	3	1	2	33.33	0.95	0.32
Bayburt	1	139	5	3	2	60.00	3.60	2.16
Hakkari	3	392	46	9	37	19.57	11.73	2.30
Kocaeli	19	581	93	12	81	12.90	16.01	2.07
Malatya	1	26	3	3	0	100.00	11.54	11.54
Mardin	6	124	19	5	14	26.32	15.32	4.03
Muş	15	4644	61	30	31	49.18	1.31	0.65
Sakarya	2	1977	43	10	33	23.26	2.18	0.51
Samsun	5	1660	95	18	77	18.95	5.72	1.08
Siirt	7	128	22	7	15	31.82	17.19	5.47
Tokat	1	17	2	1	1	50.00	11.76	5.88
Trabzon	9	1331	172	28	144	16.28	12.92	2.10
Tunceli	1	34	3	0	3	0.00	8.82	0.00
Şanlıurfa	7	377	66	15	51	22.73	17.51	3.98
Şırnak	7	392	36	9	27	25.00	9.18	2.30
Batman	3	321	50	23	27	46.00	15.58	7.17
Van	15	841	74	17	57	22.97	8.80	2.02
Bitlis	7	309	49	16	33	32.65	15.86	5.18
Konya	1	90	3	0	3	0.00	3.33	0.00
Gaziantep	4	309	47	11	36	23.40	15.21	3.56
Kars	3	774	19	6	13	31.58	2.45	0.78
Iğdır	10	2274	52	10	42	19.23	2.29	0.44
Düzce	2	55	14	1	13	7.14	25.45	1.82

**Fig. 1:**
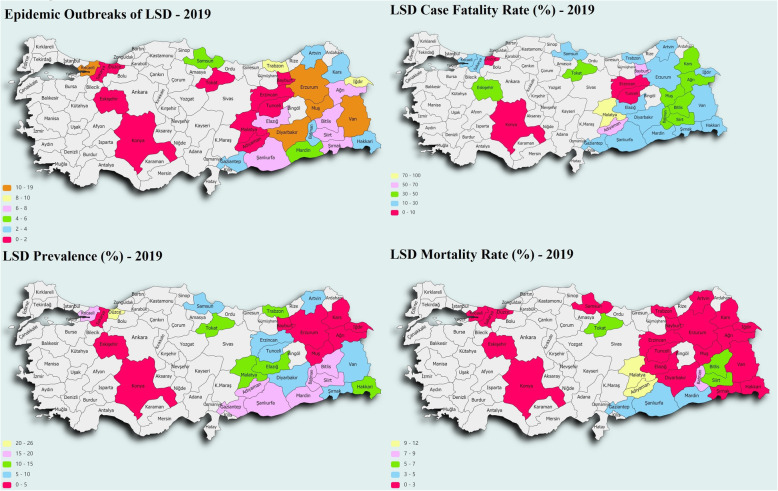
Map of epidemic outbreaks, mortality, LSD prevalence, and fatality rate by province

From Table 1, Kocaeli was the province with the most disease outbreaks (n = 19), and the month in which the most disease outbreaks are recorded was August (n = 41). The highest fatality rate (100%) and mortality (11.54) were recorded in Malatya, and the highest prevalence of the disease was observed in Düzce (25.45%). The epidemic outbreaks, mortality, LSD prevalence, and fatality rate by province are presented in Fig. 1. Although 1282 cases were recorded in Turkey during the study period, the average prevalence of the LSD epidemic was 4.51%, the fatality rate was 24.10%, and mortality was 1.09%.

When the epidemic outbreak, prevalence, fatality rate and mortality rate are examined in Fig. 1, it is seen that the disease is concentrated in the eastern and southern border provinces of Turkey.

**Fig. 2:**
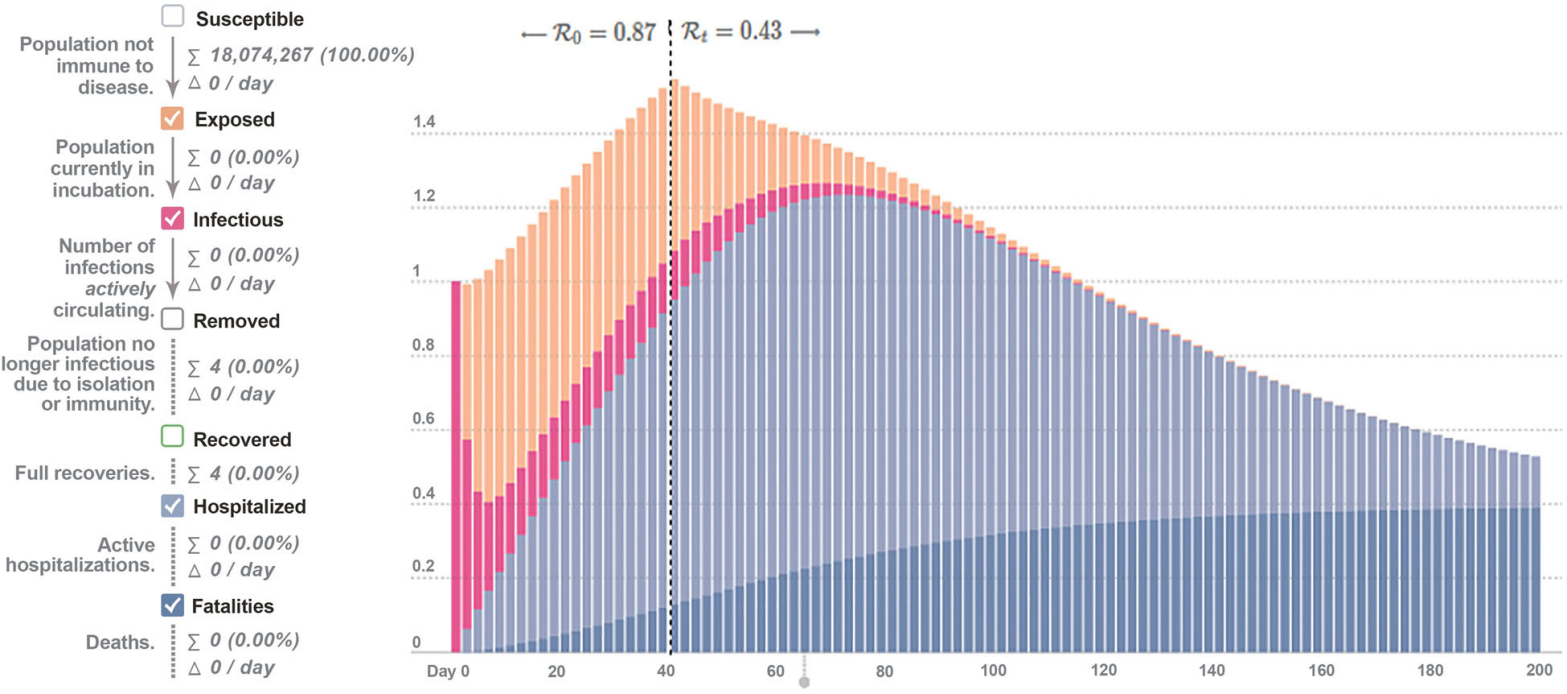
Current status of LSD infection using the SEIR model in the epidemic calculator

The current status of the disease, created using the SEIR model in the epidemic calculator with the 2019 data of LSD infection, is presented in Fig. 2.

When Fig. 2 is examined, the number of susceptible cattle population is 18.074.267. Although an infected animal infects 0.87 (R0) animal during the disease process, the rate of transmission to the animal decreases to 0.43 (Rt) after the peaked period (40th day) of the disease.

**Fig. 3:**
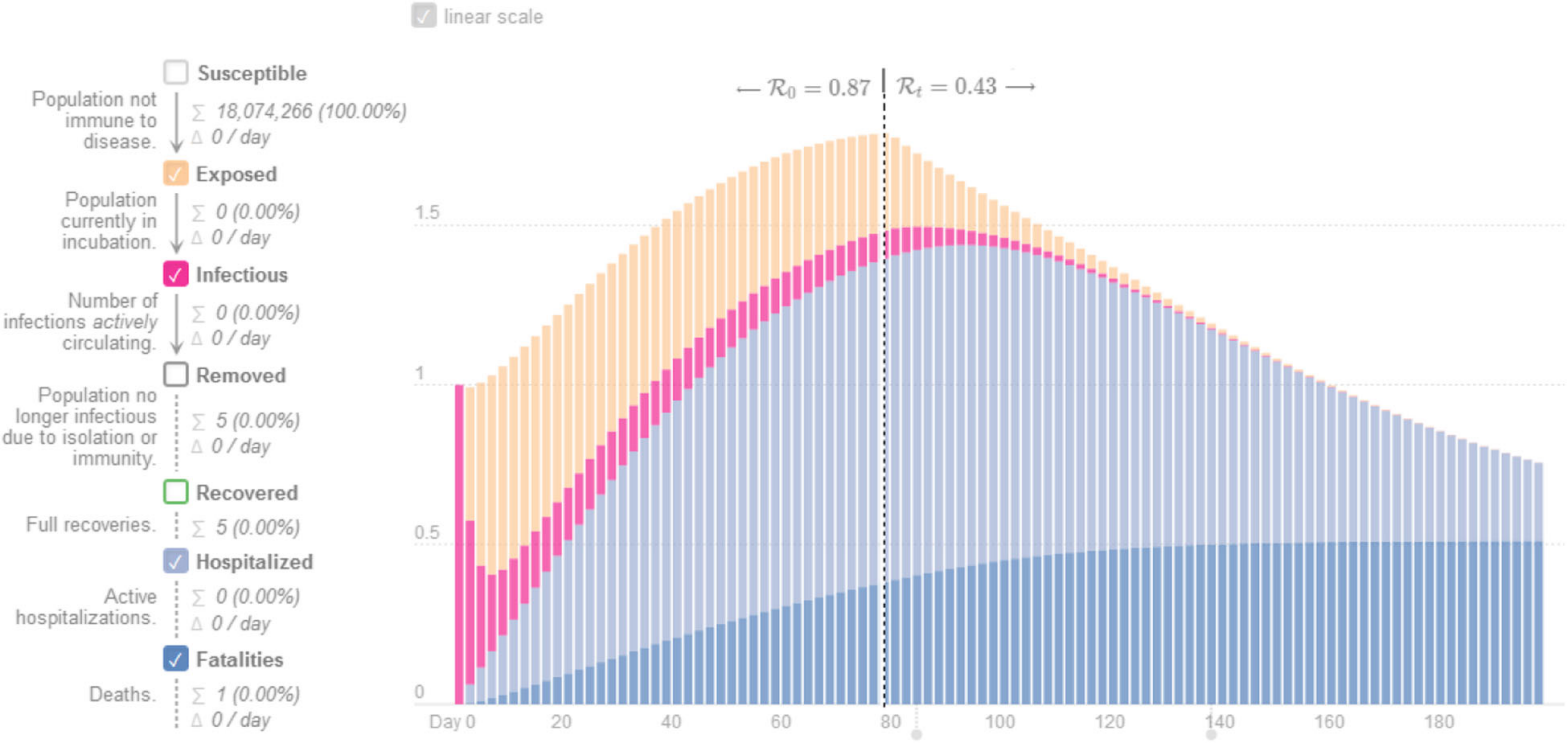
Dynamics of LSD infection using the SEIR model in case of early diagnosis

The dynamics of LSD infection modelled using the SEIR epidemic model are presented in Fig. 3. Early detection of the disease during the incubation period was observed to significantly affect the disease's peak time. If the disease was detected during the incubation period using the model, the patient would remain contagious (T_inf_) for only 2.66 days. When this situation was evaluated in terms of the disease diagnosis, a one-unit change in the incubation period caused a 2.66-fold difference in the number of patients, mortality rate, and the number of dead animals, which directly affected the economic losses caused by the disease.

**Fig. 4:**
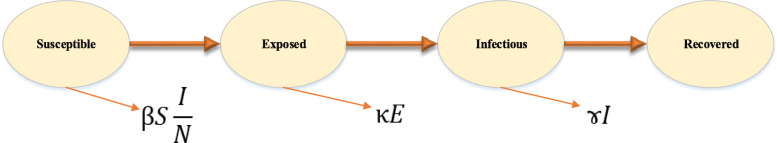
The SEIR Model

In Fig. 4, when the disease is early detected during the incubation period (T_inf_: 2.66), the dynamics of LSD infection using the SEIR epidemic model is represented.

The production losses and rates per infected animal in Turkey are presented in Table 2.

**Table 2:** Production losses due to LSD in Turkey

	Dairy cattle			Beef cattle		
Loss Type	Total Loss (US$)	Loss per Animal (US$)	Ratio (%)	Total Loss (US$)	Loss per Animal (US$)	Ratio (%)
Loss of Milk	29,918.88	46.68	5.27	-	-	-
Body weight loss	34,089.36	53.18	6.00	40.519.65	63.21	5.93
Carcass meat loss	478,978.88	747.24	84.31	621.086.07	968.93	90.84
Spontaneous Abortion and Still Birth loss	8,590.33	13.40	1.51	-	-	-
Loss of skin	16,567.06	25.85	2.92	22.089.41	34.46	3.23
Total Loss	568,144.50	886.34	100.00	683.695.13	1,066.61	100.00

The calculations made in Turkish Lira (₺) were converted into USD using the exchange rate in the relevant period (Average exchange rate: USD1= ₺ 7.83)

From Table 2, the total loss is calculated as 568,144.50 USD for dairy cattle and 683,695.13 USD for beef cattle.

## Discussion

Although Turkey’s first LSD epidemic occurred in the Kahramanmaraş province to the south of the country in 2013, the disease spread to the Southeastern and Eastern Anatolian regions until the end of 2019 (Fig. 1).

According to a study that analyzed the publicly available LSD epidemic data in the Middle Eastern countries from 2012 to 2015, the high-risk areas were countries in the Middle East's north-eastern part. On the other hand, Israel and Turkey were the most vulnerable to LSD outbreaks [3]. As a matter of fact, in the study, LSD epidemic outbreaks were intensely observed in provinces close to the border of Turkey, which corresponds to the Northeast part of the Middle East (Fig. 1).

Another study examined 611 cattle suspected of LSD infection in different farms in Turkey between July 2014 and June 2015, and reported the morbidity rate as 12.31%, the mortality rate of 6.45%, and case fatality rate of 52.4% [42]. In our study, although 1282 cases were detected in 2019, the prevalence of the LSD epidemic in Turkey was 4.51%, the fatality rate was 24.10%, and mortality was 1.09%. Compared to the studies conducted during previous years, this study's findings indicate the success of vaccinations conducted against LSD infection in Turkey. As a matter of fact, cattle are vaccinated free of charge by the state until the end of June every year within the scope of combating LSD throughout Turkey [36].

Studies conducted on non-vaccinated animals have reported a morbidity rate from LSD as 42.6% and the mortality rate as 10.2%. In contrast, in a vaccinated enterprise, the morbidity rate was 4.7% and the mortality rate was 1% [1]. Accordingly, if it is necessary to compare the vaccination costs and production losses in the fight against the disease, the preference should favor animals' vaccination. In studies conducted, the financial benefits of the vaccination program were estimated using a partial budget analysis. The net benefit per animal in Holsteins was calculated as USD 19, and the losses due to LSD decreased by 31% following vaccination [14]. The current cattle population in Turkey is vaccinated free of charge against LSD within the Agriculture and Forest ministry's scope of the vaccination program. In this context, 94.55% of the current cattle assets were vaccinated against LSD in the 2019 vaccination program [37]. As a matter of fact, the mortality rate we obtained in the study is similar to the mortality rate obtained in the vaccinated enterprises in Jordan [1]. In a study conducted on Holstein Friesian animals in Ethiopia, the incidence of LSD infection was reported as 33.9%, and the annual mortality rate was 7.43% [14]. In Jordan, the mortality rate was 1.9%, and the fatality rate was 7.5% [2]. In a study conducted on 4430 cattle in 243 herds affected by LSD infection in the central and northwestern parts of Ethiopia, the morbidity rate was calculated as 21.2%, and the mortality rate was 4.5% [28]. In another study analyzing data on 77 outbreaks of LSD in Uganda between 2002 and 2016, the morbidity rate was 4.77%, the mortality rate was 0.03%, and the case fatality rate was 0.72% [30].

The dissemination of viral diseases and disease modelling in animals and revealing the ideal protection-control programs through the developed models will enable correct vaccination schedules. On the other hand, with the outputs obtained from the developed models, when zoonotic diseases are considered, indirect and direct gains can be made in terms of public health.

It is known that LSD has an incubation period. It is assumed that we cannot infect animals that are not sick during this time. S (susceptible) represents healthy animals. They are animals that carry the E (exposed) disease but do not transmit it. I (infected) animals identify those exiting the system by death/culling. R (recovered) are animals that have recovered and become immune to the disease for a period of time. Mathematical systems whose epidemic models are used to predict the course of the disease may have critical effects on real life. Since the parameters in these models are calculated with real data, they can give an idea of ​​the transparency of the shared phenomena. There will be much more burden on economies and animal health systems when the issue is not understood in full transparency. These models, which are the basis for monitoring and predicting the spread of infectious animal diseases, can better reflect real life by using system dynamics and agent-based simulation models.

In order to reduce the R0 value to the range of 0.20-0.40 and the Rt value to the range of 0.10-0.20 for LSD, the vaccination schedule and vector struggle in the fight against the disease should be done at the right and ideal time according to the model we have created. Vaccination activities within the scope of combating LSD in Turkey are completed at the end of June. However, in order to achieve the success of the LSD protection control program, it is most critical to complete the fight against the vector before the vaccination program, taking into account regional and seasonal differences. Vector activity increases in Turkey due to the warming of the weather in June. In this context, it is recommended to complete the vaccination program in May in order to reach the ideal values in the model. By using the SEIR model in combating LSD, new and different solutions have been proposed to reduce the economic losses caused by the disease.

In simulations using the SEIR epidemic model, the detection of LSD infection during the incubation period changed the disease course, causing a decrease in production loss due to the infection (Fig. 3). In the current calculation in the study (50% dairy cattle -50% beef cattle), production losses due to LSD infection are valued at US $ 886.34 per female and the US $ 1,066.61 per male animal. The main difference in the value loss calculated in beef cattle and dairy cattle is that the economic value of carcass meat loss in male animals is 30% higher than that of females. This is a huge economic loss for Turkey, which is an importer of red meat. In addition, these losses per beef cattle and dairy cattle create economic difficulties for producers who earn their livelihood with livestock. On the other hand, even though LSD is a compensatory disease, failure to pay this compensation to producers on time increases the size of the damage. In the model framework, putting forward protection-control strategies in combating the disease will enable a decrease in the number of animals to be slaughtered and the amount of compensation to be paid.

In the research made to calculate the economic losses caused by LSD; The focus has been on direct economic losses resulting from the disease. However, it has been noticed that indirect economic losses are ignored (İnce et al.). In calculating financial losses due to LSD, only the number of animals diagnosed with LSD was calculated in the examinations made over the samples sent to the laboratories. In addition, treatment and vaccination costs have been mentioned in LSD disease, but this disease vaccination (in Turkey) is a free, untreated and notifiable disease (Şevik and İnce). Unlike other studies, in the methodology we use in calculating the economic losses caused by LSD, taking into account the gender difference in indirect losses (carcass loss, skin loss, milk loss and waste calf loss), it is handled with a comprehensive calculation method throughout Turkey.

In a study conducted in Ethiopia, over 94% of the herd owners reported LSD to be a significant problem for cattle production, and 92.2% of LSD cases affected cattle marketing. The average loss per animal in the Holstein breed due to deaths was estimated at USD 1250, that for a lactating cow was USD 216, while the diagnosis and drug cost per animal was USD 5. The average total financial loss due to an LSD epidemic at the herd level was reported to be USD 1176, and the largest loss components were deaths (USD 1000) and milk loss (USD 120) [28].

In a study aiming to measure the cost of disease and control measures applied for LSD patients in Albania, Bulgaria, and Macedonia between 2016-2017, the total cost due to the disease in the three countries was calculated to be 20.9 million Euros. (Million USD 25.45). Also, the cost per animal in the affected herds in the above countries was calculated as 539 (USD 656), 147(USD 179), and 258 (USD 314) Euros, respectively [8].

In a study conducted in Nigeria between August 2017 and January 2018, LSD-infected animals' slaughter or sale at low prices in flocks was adopted as a common coping strategy. In this case, the farmers reported that they had to sell the animals for 47% lower than their average value [24]. However, the fact that the vaccination program is at the forefront in the protection-control strategy applied in Turkey and the obligatory notification of the disease and the culling by paying the compensation does not make it possible to sell the infected animals in the market.

In Turkey, the LSD is not treated because LSD is a notifiable disease. For this reason, the loss of the enterprise owners is paid by the state. Therefore, treatment costs were not considered while calculating the production losses. However, in other studies, treatment costs have accounted for 27.9 GBP (USD 39.46) in Jordan [2] and 28.7 EUR ( USD 34.96) per animal in Albania [21].

Within the scope of the research, the lack of information about the gender of animals affected by LSD in Turkey and the cost of protection-control strategies and the effect of the vaccine used on the disease constitute the limitation of the research.

## Conclusion

The SEIR model contributes significantly to identifying animal epidemics and modelling their dynamics, control-treatment, and eradication studies. In this study, we have applied the SEIR model to determine the prognosis of LSD and have predicted that practices such as controlling animal movements, effective quarantine practices, fight against vectors, year-round vaccination, prevention of the entry of infected animals into safe herds, increasing the capacity of monitoring, and monitoring the disease will reduce the economic losses due to the disease.

In conclusion, in LSD-infected animals, significant economic losses are incurred due to reduced milk yield and body weight, spontaneous abortion, infertility, and reduced skin value. Disease control and eradication practices, such as restrictions on animal movements due to illness, vaccination activities, restriction of animal product trade, and destruction/culling of animals, are the most frequently used methods in combating the disease.

## Methods

### Data set

The materials used for this study were the data on the LSD disease outbreak in Turkey in 2019 [31]. Monthly data of 179 LSD outbreaks in 30 Turkish provinces were obtained from the OIE database. The descriptive statistics on the outbreaks were evaluated using SPSS version 20 [40].

The definition of the outbreak is expressed as seeing one or more cases in a livestock enterprise [32].

### SEIR model

The SEIR model is a compartmentalized model developed in the early 20^th^ century by Kermack and McKendrick [22]. The model was based on differential equations and simplified the mathematical modelling of infectious diseases with significant incubation periods [10]. The model is based on a homogeneous and constant population and has four compartments: Susceptible (S), Exposed (E), Infectious (I), and Recovered (R). Susceptible (S) represents the individuals entering the population by birth or immigration; Exposed (E) represents the number of individuals in contact with Infectious (I) during the incubation period; Infectious (I) represents the number of infected individuals; and Recovered (R) is the number of individuals recovered from the infection [47]. The individuals in the model progress successively along with these compartments.

An SEIR model is evaluated in two parts. The first part is the transmission dynamic, including population inputs, basic reproductive number (*R*_0_), and transmission time.

Population inputs (size of the population and number of initial infections), basic reproductive number (*R*_0_), and transmission times (length of the incubation period, the duration for which the patient is infectious) are the dynamics of the model, and are calculated based on the following differential equations for *S (t)*, *E (t)*, *I (t)*, *and R (t)*:


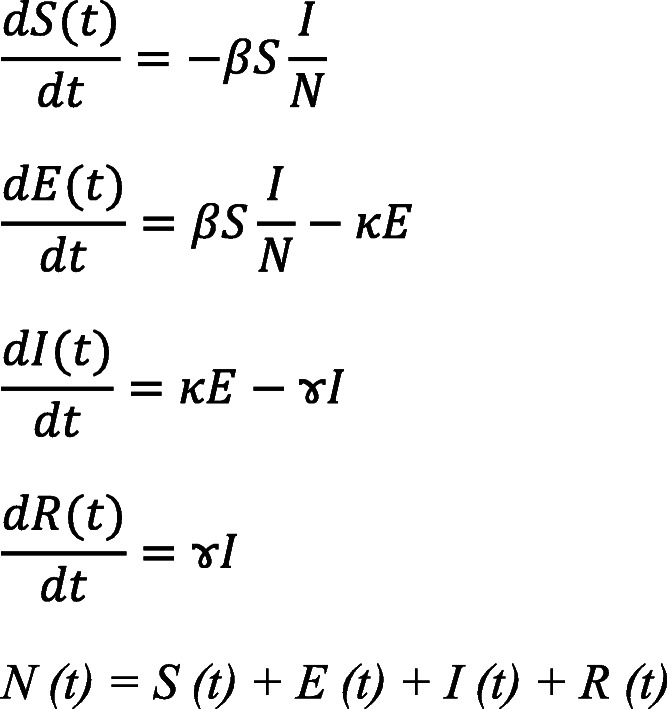


where the parameters *β*, *κ*,and *ɤ*_*t*_) and the number of secondary infections produced by each infected individual, known as the basic reproduction number (*R*_0_) are determined. The above parameters represent the proportion of a specific population among the total population and are calculated as described by Legrand [23] and Diaz [10] as follows:


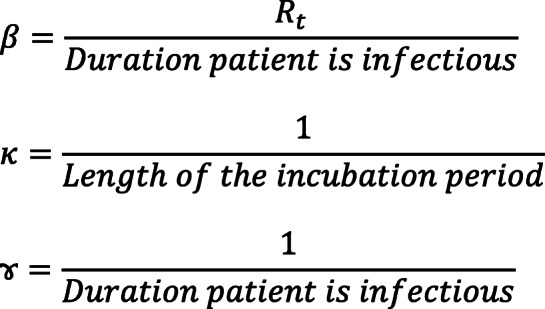


The second part is the clinical Dynamics, comprising the Mortality Statistics (Case fatality rate and time from the end of incubation to death), Recovery Time (length of hospital stay and recovery time for mild cases), and Care Statistics (Hospitalization rate and time to hospitalization).

The transmission and clinical dynamics of LSD are presented in Table 3.

**Table 3:** Parameters in the LSD dynamics model.

Dynamics		Inputs	Values	References
Transmission dynamics	Population Inputs	Size of population	18070500	[25]
		Number of initial infections	1	Expert Opinion
		Basic Reproduction Number R_0_ Measure of contagiousness: the number of secondary infections each infected individual produces.	0.87	[12]
	Transmission Times	Length of the incubation period	28 (day)	[31]
		Duration patient is infectious	35 (day)	[31]
Clinical Dynamics	Mortality Statistics	Case fatality rate	9.52 (%)	[18]
		Time from end of incubation to death	4-6 (weeks)	[15]
	Recovery Times	Recovery Times Length of hospital stay	4-6 (weeks)	[15]
		Recovery time for mild cases	4-6 (weeks)	[15]
	Care statistics	Hospitalization rate	71.48(%)	Calculated From Data Set
		Time to hospitalization	30-45 (day)	Expert Opinion

An online SEIR model simulator was applied to model the LSD infection and determine the disease dynamics [17].

### Calculation of production losses

In the calculation of animal production losses caused by LSD, 50% of sick animals were evaluated as dairy cattle and 50% as beef cattle. Since the information of the sex of the dead animals could not be obtained from in the analyzed data set, the loss of body weight, carcass, and skin was considered to calculate losses per infected animal in the beef cattle, while the loss of milk yield, spontaneous abortion, and stillbirth were added to the above losses in the dairy cattle. The effect of LSD on buffaloes was not calculated as the buffalo population in Turkey is negligible.

Production losses due to LSD infection [14, 42, 46] are covered in the analysis of economic loss, the items and calculation procedures for which are presented in Table 4. While calculating the production losses, loss of milk production, live weight, carcass meat, spontaneous abortion and stillbirth, and weight of skin were estimated. It was assumed that the losses caused by LSD did not affect the market of animal products. Economic costs and losses due to the use of fertilizers, biosecurity measures, and insecticide applications, especially the losses due to fertility, were overlooked. The missing components and calculation procedures are presented in Table 4.

**Table 4:** Estimation of LSD-related production losses in cattle

Missing Component	Calculation method:
Loss of milk production (I)	Number of Animals * Decrease in Daily Milk Yield (L) * Average Number of Days * Average Milk Price
Body weight loss (II)	Number of Animals * Average Carcass Weight (Kg) * CA Decrease Rate (%) * Average Live Beef Price
Carcass meat loss (III)	Number of Animals * Average Carcass (Kg) * CA Decrease Rate (%) * Average Carcass Price * Carcass Yield
Spontaneous Abortion and Still Birth loss (IV)	Number of Animals * Spontaneous Abortion and Still Birth Rate (%) * Average Calf Price
Loss of skin (V)	Number of Animals * Average Skin Price
Total Loss	(I+II+III+IV+V)

The details and sources of technical and financial data used in the analysis are presented in Table 5.

**Table 5:** Epidemiological and production parameters used in the estimation of LSD-related production losses in cattle in Turkey

Parameters used in the analysis	Value	Reference
*Technical Parameters*		
Total number of animals (head) with LSD	1282	[31]
Milk Loss (L/head/day)	5.3	[42]
Female Carcass Weight (Kg/head)	387	[4, 43]
Male Carcass Weight (Kg/head)	460	[4, 43]
Live Weight Reduction Rate (%)	6.22	[14]
Female Carcass Yield (%)	55	[41]
Male carcass yield (%)	60	[41]
Spontaneous Abortion and Still Birth Rate (%)	3	[46]
*Financial Parameters*		
Milk price (TL/L)	2.3	[48]
Carcass Meat Price (TL/kg)	29.33	[16]
Live Beef Price (TL/kg)	17.31	[16]
Calf Price (TL/head)	3.500	[11]
Skin Price (TL/piece)	4.5	[19]

## Data Availability

The datasets used and/or analysed during the current study are available from the corresponding author on reasonable request.

## Abbreviations

LSD: Lumpy Skin Disease-LSD
SEIR: Susceptible (S), Exposed (E), Infectious (I), and Recovered (R)
USD: United States Dollar

## References

[1] Abutarbush SM (2014). Efficacy of vaccination against lumpy skin disease in Jordanian cattle. Vet Rec.

[2] Abutarbush SM, Ababneh MM, Al Zoubi IG, Al Sheyab OM, Al Zoubi MG, Alekish MO, Al Gharabat RJ (2015). Lumpy skin disease in Jordan: disease emergence, clinical signs, complications and preliminary-associated economic losses. Transbound Emerg Dis.

[3] Alkhamis MA, VanderWaal K (2016). Spatial and temporal epidemiology of lumpy skin disease in the Middle East, 2012–2015. Front Vet Sci.

[4] Alpan O, Aksoy AR (2015). Cattle breeding and fattening, 7th edition. Favori Printing & Publishing San.

[5] Balenghien T, Chalvet-Monfray K, Bicout DJ, Sabatier P (2005). Modelling and determination of the transmission contact rate for contagious bovine pleuropneumonia. Epidemiol Infect.

[6] Balenghien T, Chalvet-Monfray K, Lesnoff M, Thiaucourt F, Sabatier P, Bicout D (2004). Time-delay dynamics for contagious bovine pleuropneumonia. Acta Biotheor.

[7] Caglar O. (2019). Spatial Analysis of A Viral Agent First Time Appeared In Turkey By Geographic Information System: Cattle Noduler Exhantem (Lumpy Skin Disease-LSD), Hacettepe University Graduate School of Health Sciences Epidemiology Program Master of Science Thesis, Ankara. Access: http://www.openaccess.hacettepe.edu.tr:8080/xmlui/handle/11655/9163. Accessed: 02.03.2021.

[8] Casal J, Allepuz A, Miteva A, Pite L, Tabakovsky B, Terzievski D, Beltrán-Alcrudo D (2018). Economic cost of lumpy skin disease outbreaks in three Balkan countries: Albania, Bulgaria and the Former Yugoslav Republic of Macedonia (2016-2017). Transbound Emerg Dis.

[9] Davies FG (1991). Lumpy skin disease of cattle: A growing problem in Africa and the Near East. World Anim Rev.

[10] Diaz P, Constantine P, Kalmbach K, Jones E, Pankavich S (2018). A modified SEIR model for the spread of Ebola in Western Africa and metrics for resource allocation. Appl Math Comput.

[11] DSYMB (2020). Price statistics. Cattle Breeders Central Union of Turkey.

[12] Calistri P, DeClercq K, Gubbins S, Klement E, Stegeman A, Gogin A, EFSA 2019. European Food Safety Authority (2019). Lumpy skin disease: III. Data collection and analysis. EFSA J.

[13] EFSA 2015 (European Food Safety Authority) AHAW Panel (EFSA Panel on Animal Health and Welfare). Scientific Opinion on Lumpy Skin Disease. EFSA J. 2015;13(1):3986. http://www.efsa.europa.eu/en/efsajournal/doc/3986.pdf.

[14] Gari G, Bonnet P, Roger F, Waret-Szkuta A (2011). Epidemiological aspects and financial impact of lumpy skin disease in Ethiopia. Prev Vet Med.

[15] Gazioğlu A (2016). Sığırların Nodüler Ekzantemi (Lumpy Skin Disease). Türkish J Agric Nat Sci.

[16] GDMMB (2020). General Directory of Meat and Milk Board. Weekly meat price bulletin. https://www.esk.gov.tr/; Accessed: 06.10.2020.

[17] Goh G. (2020). Epidemic Calculator. Available online: http://gabgoh.github.io/COVID/ accessed: 25.05.2020

[18] Ince OB, Türk T (2019). Analyzing risk factors for lumpy skin disease by a geographic information system (GIS) in Turkey. J Hellenic Vet Med Soc.

[19] İTB (2020). Daily Stock Market Bulletin. Izmir Commodity Exchange. Access: https://itb.org.tr/GunlukBultenler/1-tescil-bulteni. Accessed:19.10.2020

[20] Kahsay T, Negash G, Hagos Y, Hadush B (2015). Pre-slaughter, slaughter and post-slaughter defects of skins and hides at the Sheba Tannery and Leather Industry, Tigray region, northern Ethiopia. Onderstepoort J Vet Res.

[21] Karalliu E, Boçi R, Hatia V, Prifti V, Keçi R, Manaj B, Koleci X (2017). A case study of lumpy skin disease outbreak in Rrapez. Lushnje. Albanian J Agric Sci.

[22] Kermack WO, McKendrick AG (1927). A contribution to the mathematical theory of epidemics. Proceedings of the royal society of london. Series A, Containing papers of a mathematical and physical character.

[23] Legrand J, Grais RF, Boelle PY, Valleron AJ, Flahault A (2007). Understanding the dynamics of Ebola epidemics. Epidemiol Infect.

[24] Limon G, Gamawa AA, Ahmed AI, Lyons NA, Beard PM (2020). Epidemiological Characteristics and Economic Impact of Lumpy Skin Disease, Sheeppox and Goatpox Among Subsistence Farmers in Northeast Nigeria. Front Vet Sci.

[25] MAF (2020). Ministry of Agriculture and Forestry. Livestock data. https://www.tarimorman.gov.tr/sgb/Belgeler/SagMenuVeriler/HAYGEM.pdf, Accessed: 15.06.2020.

[26] Megersa B, Biffa D, Abunna F, Regassa A, Bohlin J, Skjerve E (2012). Epidemic characterization and modeling within herd transmission dynamics of an “emerging trans-boundary” camel disease epidemic in Ethiopia. Trop Anim Health Prod.

[27] Mohammed A, Abdulai A, Birteeb PT, Husssein SMA (2018). Major causes of organ and carcass condemnations of cattle and their associated financial loss at the Tamale abattoir, Ghana. UDS Int J Dev.

[28] Molla W, de Jong MC, Gari G, Frankena K (2017). Economic impact of lumpy skin disease and cost effectiveness of vaccination for the control of outbreaks in Ethiopia. Prev Vet Med.

[29] Nielsen JP, Larsen TS, Halasa T, Christiansen LE (2017). Estimation of the transmission dynamics of African swine fever virus within a swine house. Epidemiol Infect.

[30] Ochwo S, VanderWaal K, Munsey A, Ndekezi C, Mwebe R, Okurut ARA, Mwiine FN (2018). Spatial and temporal distribution of lumpy skin disease outbreaks in Uganda (2002–2016). BMC Vet Res.

[31] OIE (2020). https://www.oie.int/fileadmin/Home/eng/Animal_Health_in_the_World/docs/pdf/Disease_cards/LUMPY_SKIN_DISEASE_FINAL.pdf. Accessed: 05.01.2021.

[32] OIE (2019). https://www.oie.int/fileadmin/Home/eng/Health_standards/tahc/current/glossaire.pdf. Accessed: 05.01.2021.

[33] Park AW, Wood JLN, Daly JM, Newton JR, Glass K, Henley W, Grenfell BT (2004). The effects of strain heterology on the epidemiology of equine influenza in a vaccinated population. Proceedings of the Royal Society of London. Series B: Biol Sci.

[34] Roda WC, Varughese MB, Han D, Li MY (2020). Why is it difficult to accurately predict the COVID-19 epidemic?. Infect Dis Modelling.

[35] Rorres C, Pelletier ST, Smith G (2011). Stochastic modeling of animal epidemics using data collected over three different spatial scales. Epidemics.

[36] RTMFAL (2019a). Republic of Turkey Ministry of Food, Agriculture and Livestock. Fighting Animal Diseases and Animal Movement Control. https://samsun.tarimorman.gov.tr/Belgeler/2019/2019_Hayvan_Hastaliklari_ile_Mucadele_GENELGE.pdf. Accessed: 13.02.2021.

[37] RTMFAL (2019b). Republic of Turkey Ministry of Food, Agriculture and Livestock. Livestock vaccination program of Turkey. https://samsun.tarimorman.gov.tr/Belgeler/2019/2019_yili_asilama_programi.pdf. Accessed: 13.02.2021.

[38] Savini L, Candeloro L, Conte A, De Massis F, Giovannini A (2017). Development of a forecasting model for brucellosis spreading in the Italian cattle trade network aimed to prioritise the field interventions. PLoS One.

[39] Simon A, Tardy O, Hurford A, Lecomte N, Bélanger D, Leighton P. Dynamics and persistence of rabies in the Arctic. Polar Res. 2019;38:3366. 10.33265/polar.v38.3366.

[40] SPSS (2013). SPSS for Windows, Version 20.

[41] Şentürk B, Akçay A, Sarıözkan S (2020). Estimating the cost of bovine tuberculosis at the public and farm levels: The case of Samsun Province, Turkey. Vet J Mehmet Akif Ersoy Univ.

[42] Şevik M, Doğan M (2017). Epidemiological and molecular studies on lumpy skin disease outbreaks in Turkey during 2014–2015. Transbound Emerg Dis.

[43] Tıknazoğlu B (2010). Cattle breeding, Samsun Provincial Directorate of Agriculture, Farmer Education and Extension Branch Publication, Samsun.

[44] Tuppurainen E, Alexandrov T, Beltrán-Alcrudo DJFAP, Manual H (2017). Lumpy skin disease field manual-A manual for veterinarians. FAO Ani Prod He Man.

[45] Uyar A, Yener Z, Yıldırım S, Keleş ÖF (2015). A lumpy skin disease (nodular exanthem) case in a holstein cow. FÜ Sağ Bil Vet Derg..

[46] Vorster JH, Mapham PH (2008). Pathology of lumpy skin disease. Livest Hilth Prod Rev..

[47] Zhou X, Cui J (2011). Analysis of stability and bifurcation for an SEIR epidemic model with saturated recovery rate. Commun Nonlinear Sci Numer Simul.

[48] Ulusal Süt Konseyi (2020). 1 Liter of raw milk cost by regions. Access: https://ulusalsutkonseyi.org.tr/bolgelere-gore-1-litre-cig-sut-maliyeti-1637/. 460. Accessed:20.9. 2020

